# Exploring Iranian women’s perceptions and experiences regarding cervical cancer-preventive behaviors

**DOI:** 10.1186/s12905-018-0635-8

**Published:** 2018-08-31

**Authors:** Maryam Khazaee-Pool, Fatemeh Yargholi, Fatemeh Jafari, Koen Ponnet

**Affiliations:** 1 Department of Health Education and Promotion, School of Public Health, Zanjan University of Medical Sciences, Zanjan, Iran; 2 Department of Public Health, School of Public Health, Zanjan University of Medical Sciences, Zanjan, Iran; 3 Department of Communication Sciences, imec-mict-Ghent University, Ghent, Belgium

**Keywords:** Perception, Preventive behaviors, Cervical cancer, Qualitative study

## Abstract

**Background:**

Preventive behaviors regarding cervical cancer are essential for women’s health. Even though many studies have addressed women’s knowledge and attitudes toward cervical cancer, little information is available about their experiences of cervical cancer-preventive behaviors. Thus, the aim of this study is to explore the perceptions and experiences of Iranian women regarding cervical cancer-preventive behaviors.

**Methods:**

This study used a qualitative approach and was conducted in Zanjan, Iran. Participants included 27 women, aged 20–60 years, with no previous history of cervical cancer symptoms or diagnosis. Data were obtained through semi-structured in-depth interviews and focus group discussions. Inductive qualitative content analysis was employed to converge and compare themes through participant data.

**Results:**

The following six main themes emerged from the analysis: attitudes toward cervical cancer and preventive behaviors, preventive behaviors’ concept, self-care, religion and culture, perceived social support, and awareness about cervical cancer and preventive behavior. The findings revealed that several women had misconceptions about cervical cancer and were even superstitious about the causes of it. Fear, shame, and embarrassment were reasons for not undertaking cervical cancer screening. Cervical cancer was also linked to worries about decreased marital satisfaction, sexuality, and femininity. However, religion was considered a positive factor to conducting cancer-preventive behaviors.

**Conclusions:**

This study showed that improving knowledge about the causes of cervical cancer, increasing awareness of the potential consequences of it, and creating positive attitudes toward screening behavior might encourage Iranian women to perform cervical cancer-preventive behaviors.

## Background

Cervical cancer is the second most prevalent cancer in the world among young females, and it is the leading cause of cancer mortality in women [1, 2]. In 2012, around 528,000 women were diagnosed with cervical cancer, and about 276,000 women died of cervical cancer, with more than 70% of these women living in low-income countries [3]. In Iran, cervical cancer is among the five most common cancers and ranks fourth among cancers diagnosed in females. Although the incidence of cervical cancer in Iran is still moderately low (2.2 per 100,000 women annually), and the prevalence of human papillomavirus infection is 7% among healthy Iranian women, the number of patients with late diagnosis of cervical cancer is increasing, and the mortality rate is high [4]. However, there are no reliable statistics about the incidence and mortality rates of cervical cancer in Iran due to the lack of an appropriate cancer recording system [4].

There are several risk factors of cervical cancer. Next to human papillomavirus (HPV) [5], some behavioral factors that can be modified or avoided may play an important role in the increased risk for developing cervical cancer. Among those factors are not eating enough fruits and vegetables [6], being overweight [7], long-term use of oral contraceptives (birth control pills) [8], intrauterine device (IUD) use [9], having multiple full-term pregnancies [10], exposure to diethylstilbestrol (DES) [11], low physical activity [12], smoking [13], having multiple sexual partners [14], and human papilloma virus infection [15]. These lifestyle factors are not only important risk factors for developing cervical cancer but also impact health outcomes and quality of life [16, 17]. Thus, it can be argued that the most beneficial and effective means of reducing cervical cancer burden and mortality is prevention and early detection [16, 17]. Furthermore, in order to identify all possible risk factors, it is vital for women to receive a regular Pap smear test to diagnose cervical cancer early.

It has been advised that the best way to prevent cervical cancer is for women to change their lifestyle and receive regular Pap tests. Lifestyle change can help to decrease the risk of cervical cancer to a large scale [18]. Previous studies have shown that lifestyle modifications such as eating fruits and vegetables [6], maintaining a healthy weight [7], regular physical activity [12], stopping OCP use [9], avoiding exposure to HPV and getting an HPV vaccine [15], and quitting smoking [13] may help to decrease the risk and prevent cervical cancer. Furthermore, screening behavior is considered useful for early detection in the pre-malignant phase and reduced mortality on account of cervical cancer [19]. However, the guidelines for applying this method are not consistent and differ in various countries [20–23]. The observed differences in cervical screening guidelines are due to limited resources and, in some cases, sociocultural factors. For example, in the U.S., cervical screening is routinely suggested for women between the ages of 21 to 65, regardless of age at first sexual intercourse or other risk behaviors [22, 24]. For healthy women aged 21–29, a Pap smear should be performed every 3 years, regardless of HPV vaccination status [25]. There is, however, no accessibility for providing a Pap smear for women in some poor resource countries [26–29]. Additionally, different sociocultural evidences are well documented in the adherence to cervical cancer screening programs [20–23, 25]. For instance, a study has demonstrated that immigrant women with different sociocultural backgrounds living in developed countries considered regular Pap tests less than native women [30]. Furthermore, in some countries women hold the belief that if someone tests her uterus, she might develop cervical cancer [31, 32].

All women aged 35–54 are at risk of developing cervical cancer. Based on the national population-based screening program in Iran, a Pap smear test must be done for all married Iranian women after marriage, and a Pap test must be done three years after marriage for women. After three normal and accurate Pap smear tests and no other risks, it could be repeated every 3 years [33]. In spite of the fact that a Pap smear test can be performed free of charge in Iranian health care centers, only a small percentage of eligible Iranian women underwent this test, with percentages ranging from 14.8 to 28.3%. Several reasons account for this low percentage, including, among others, not knowing the importance of the test, the test is difficult to access, being afraid about the results of the test, and being embarrassed to undergo the test [34–36].

To this end, one might conclude that lifestyle changes and screening behavior are key matters in cervical cancer prevention programs. Yet it is essential to consider that none of the behaviors discussed could be considered the best behavior for cervical cancer prevention. Therefore, the present study aimed to explore Iranian women’s experiences of cervical cancer-preventive behaviors and develop more insight on the topic. It was expected this part of the study would contribute to the current literature from a country where lifestyle change interventions and cervical cancer screening programs are lacking and women live in a crossroad of customs, cultures, religions, and modernity.

## Methods

### Design and participants

A qualitative approach was used in this work. Participants for the present study were recruited from a health care center affiliated with Zanjan University of Medical Sciences. In order to gain a different viewpoint, a purposive sampling method was applied to insure that women with varying levels of socioeconomic background, age, educational level, job, and marital status were present in this work. At the beginning of interviews, women were informed about the aim of the study.

This study consists of in-depth interviews and focus group discussions. Data was derived from the sum of secondary analysis from a qualitative research conducted by Yargholi in which 15 women from Khoramdareh [Zanjan] about the concept of cervical cancer-preventive behaviors and its effective components were explored [37], and interview with 12 new volunteered women from Zanjan.

i. Semi-structured interviews

Overall, the 19 women (15 women’s from Yargholi’ study [37] and four new women) volunteered to participate in semi-structured interviews. Sampling was continued until data saturation was reached, that is no new codes were found in the data. At the beginning of each interview, all women were asked about demographic information. Thereafter, participants were asked to discuss their knowledge, perceptions, and behaviors regarding cervical cancer and prevention. They were also questioned about social, cultural, and environmental factors that might have influenced their behaviors. Then, based on each of the stated behaviors, questions were asked such as, “What do you do to prevent cervical cancer?”, “What behaviors might prevent cervical cancer?”, “Which factors are associated with behaviors that might prevent cervical cancer?”, and “How do you perform preventive behaviors?” After each question, participants were invited to explain more about what they had reported. For example, they were questioned, “What do you mean?” and “Explain more” for a deeper consideration of women’s experiences regarding cervical cancer prevention. The interviews were performed at the women’s homes or at a convenient public place. Each interview lasted 60–120 min.

ii. Focus group discussions

The volunteered women who participated in semi-structured interviews were asked to participate in focus group discussions (FGDS). Four of them agreed to be participated in FGDS. In the next part, the eight other new volunteered women agreed to participate in FGDS. Overall, FGDS among 12 women were performed to explore the topic. Sessions were performed in places that were convenient and available for participants. Each session lasted for about 2 h.

### Data analysis

Inductive qualitative content analysis (a bottom-up method to explore the data) was employed based on the Graneheim and Braun methods to converge and compare themes through participant data. Themes were clustered according to participants’ views and experiences. In this method, the researcher recognized the themes based on the primary codes and categories. As such, the units of analysis are the entire interviews [38, 39]. Data analysis started throughout the data-gathering process. Each FGD and individual interview was transcribed literally and analyzed before the next FGD or interview was accomplished. Thorough understanding of the data was reached by frequently reading the transcriptions. All transcripts were imported into the MAXQDA software.

### Ethics

Participants completed informed written consent. All interviews were audiotape and transcribed with participant’s consent.

### Validation

For validation in present study, interview transcripts and the derived codes from each of the interviews were offered to the women and their opinions about the meaning of the codes were questioned; if they stated differing opinions, their corrective statement were made. Besides the study team, the text of the interviews was offered to some of the investigators who were not participated in present study as external observers in order to check the coding process accuracy.

## Results

In all, 27 women aged 20 and over without cervical cancer took part in the study. The participants’ demographic characteristics are presented in Table 1. Overall, six major themes emerged from the analysis: attitudes toward cervical cancer and preventive behaviors, preventive behaviors’ concept, self-care, religion and culture, perceived social support, and awareness about cervical cancer and preventive behavior*.* More information on themes and categories can be found in Table 2. In the following section we describe participants’ experiences.

**Table 1:** Socio demographic characteristics of participants (*n* = 27)

	Number	Percentage
Age group		
≤ 30	3	11.11
31- 40	17	62.97
≥ 41	7	25.92
Education		
Under diploma	5	18.52
Diploma	6	22.22
Upper	16	59.26
Marital status		
Single/divorced/widowed	6	22.22
Married	21	77.78
Occupation		
Employed	8	29.70
Housewife	19	70.30

**Table 2:** Main themes and categories

Main themes	Categories
Attitudes toward cervical cancer and preventive behaviors	Superstitious beliefs and misconceptions
	Fear about cervical cancer screening
	Fear about the consequences of cervical cancer
Concept of preventive behavior	Challenging in understanding the concept of preventive behaviors
	Pros and cons of preventive behavior
Self-care	Healthy lifestyle
	Stress management
	Medical checkups
Religion and culture	Islamic laws
	Cultural norms
	Faith in God
Perceived social support	Social support
	Informational support
	Financial support
Perceptions of cervical cancer risk	Inaccurate risk perception
	Insufficient education
	Misinformation

### Attitudes toward cervical cancer and preventive behaviors

Attitudes toward cervical cancer and preventive behaviors contained three categories about beliefs and feelings, namely superstitious beliefs and misconceptions, fear about cervical cancer screening, and fear about the consequences of cervical cancer (e.g., fear of reduced marital satisfaction, shame, illness, and negative self-image).

#### Superstitious beliefs and misconceptions about cervical cancer

Most participants had superstitious and negative beliefs toward cervical cancer and the link between cervical cancer and death. Some women who believed that nothing could be done to prevent cervical cancer had no motivation to apply preventive behavior like clinical care. They were even afraid of clinical care and believed that speaking about cervical cancer might put them at risk of getting cervical cancer. As one woman said:

*“I don’t want to talk about cervical cancer at all … Talking about cervical cancer brings bad chance. When speaking about this [cervical cancer], I will get it ...”* (Participant 20; 48 years old, married).

Another superstitious belief was that *Hesadat*, or the “evil eye,” could cause cervical cancer. *Hesadat* means jealousy or people wishing disease onto others who have what they don’t. Some participants also believed that, due to *Hesadat* inflicted on other people, cervical cancer might occur as a disease. *Bezanbetakhteh* is another word used in Iran to express when someone feels that other people are envious and have used Hesadat. *Bezanbetakhteh* means “God remove its evil effect” and is used to remove the bad effects of *Hesadat*. As one woman expressed:

*“They believe that we can catch cervical cancer due to Hesadat … people look at others … They use hesadat … With regard to cancer, they believe the cause is Hesadat ... Since they didn’t say “Bezanbetakhteh” [God remove its evil effect].”* (Participant 19; 32 years old, widowed).

#### Fear about cancer screening

Fear was a main concept that was linked to different perspectives about screening for cervical cancer. Most participants formulated some emotional replies in which they linked fear to cervical cancer. These included the fear of suffering from and dying of cervical cancer, worries about Pap test results, and pain from performing Pap tests. Some of them expressed that the fear of getting cervical cancer and, as a result, dying, was the main motivating force for them to perform a Pap smear test. Some women expressed that worries and fears about Pap smear test results were the main barriers to performing screening.

*“At this time, for sure, it is awful. If the results from the cervical cancer clinic confirm that I have cancer, what can I say? Then I suddenly will become depressed. And I can’t be sure what I can do or how I will face the reality. If it is true, what about my future life? What about my husband … What about my daughter?”* (Participant 24; 33 years old, married).

Women were also afraid of the screening approach and the related pain in the screening procedure. Some participants reported specific physical and emotional pain experienced due to an aggressive doctor and a hurried or painful Pap smear conducted by an untrained doctor, and these experiences negatively impacted their willingness to be screened once more. These experiences emphasize the influence of some uncooperative methods in negative experiences and undermine the willingness to be screened.

*“I just performed a Pap smear four years ago … because I found it is very painful, I decided not to perform it anymore … I think the doctors should be more gentle … I mean, I did it in [the clinic], and the physician pulled my uterus too hard. It was intolerable … it was awful, so I do not desire to perform it once again.”* (Participant 16; 32 years old, married).

A few sources of worries and fears came specifically from village women. One considerable case was the worry and fear about the cleanliness and health of the medical tools.

*“I’m afraid of the speculum … It is not appropriately cleaned. It is not respectable. A healthy woman might catch genital disease through the speculum.”* (Participant 26; 34 years old, single).

*“The doctor must explain to us before starting the Pap smear if the tool is disposable or if it is not, whether it has been well cleaned.”* (Participant 17; 33 years old, married).

#### Fear about the consequences of cervical cancer

Some women believed the most social consequence of getting cervical cancer was losing sexual appetite and marital satisfaction. For them, the invasive nature of the disease made it similar to the body being swallowed, which is an especially disturbing fate in Iranian culture. Women believed that cervical cancer would lead to the loss of the uterus and ovaries. They believed that loss of the reproductive organs would disable their sexual and fertility functions and would finally undermine their marital relationships. As one 32-year-old screened woman said:

*“Losing reproductive* organs *[hysterectomy] impacts family life, day-to-day life, and the marital relationship. This most valuable body part [the uterus] makes you brilliant and is a sign of your femininity. If you develop cervical cancer and it’s crucial to remove the reproductive organs, maybe you will lose both the uterus and ovaries. Whenever this happens, you will have none of the distinguished features of a woman anymore … It might lead to divorce and the loss of marital relations.”* (Participant 19; 32 years old, widowed).

Furthermore, some participants said that it is shameful to show their private parts to a doctor or even talk about their sexual problems or diseases. Most women also expressed that cervical cancer is a very shameful disease compared with other diseases because it infers that a woman is guilty of corrupt sexual activities, which are equal to promiscuity. One woman who had not been screened explained her worries as follows:

*“A woman with cervical cancer is observed as a bad person. She is viewed as filthy or raffish. Leaving aside cervical cancer, even something like women’s genital infections can provoke superstitions, especially in the suburbs. A woman may feel that she is under fastidiousness due to having cervical cancer.”* (Participant 20; 48 years old, married).

The women’s perspectives of the cervical cancer consequences were also influenced by their own experiences with family or friends who had cervical cancer. Although most women described cervical cancer as a fatal and non-preventive disease, a few had an optimistic opinion related to cervical cancer. A screened woman expressed it this way:

*“If a developing cervical cancer will be diagnosed in me, I would reject doing the treatment like chemotherapy, since the last outcome is alike and is death ... One of my best friends died because of cervical cancer, although she had performed Pap smear tests regularly and was also a healthy swimming instructor for 14 years. I believe that cervical cancer is a fatal disease that cannot be prevented.”* (Participant 17; 33 years old, married).

Some of them described the negative effects and self-image of cervical cancer on a person’s physical health and appearance. Some women also believed that cervical cancer is equal to the loss of their femininity. A participant said:

*“I dislike hearing the word cervical cancer. In my opinion, for a lady, cervical cancer is the loss of feminine identity. I believe that a woman with cervical cancer is not attractive any more, nor feminine. Cervical cancer is equal to death for a woman. It is the end of a good marital life. I am very afraid of getting cervical cancer.”* (Participant 24; 33 years old, married).

A woman stated that living without a uterus or genital organs is meaningless. She said:

*“I know the uterus and genital organs are very vital to women. Due to their maternal role, they [genital organs] are essential for pregnancy and childbirth. Furthermore, women with a uterus and all genital organs look flawless and perfect. A woman can never be complete and beautiful without genital organs. I rather wish to be dead than to live without my uterus and genital organs.”* (Participant 17; 33 years old, married).

In sum, we found that superstitious beliefs and misconceptions, fear about cervical cancer screening, and fear about the consequences of cervical cancer were very important components of women’s attitudes toward cervical cancer and prevention.

### Concepts of preventive behavior

One of the elicited themes in the current study was the concept of health, including three different categories, namely challenges in understanding the concept of preventive behaviors and the pros and cons of preventive behaviors.

#### Challenges in understanding the concept of preventive behaviors

The concept of preventive behavior was challenging to understand. A number of women specifically mentioned the lack of symptoms as a main reason for not taking preventive behaviors like a Pap smear.

*“My health is nice … There is nothing to be concerned about regarding my health and life … you know … for me everything is okay.”* (Participant 20; 48 years old, married).

Some informants explained their worries and confusion about the medical examinations for abnormal uterine bleeding. Women frequently misunderstood the purpose of pap smear as a preventive behavior and this extended to the idea of screening for latent disease. Additionally, there were a few examples in which women delayed the Pap tests until some symptoms appeared.

“*In my view, Pap smear tests cannot find out cervical cancer signs ... I was screened four times by a Pap test, but all the times I was told that everything was okay. But when I went to have the ultrasound, they told me that I had fibroids due to bleeding too much …”* (Participant 23; 31 years old, married).

*“A young woman like me, I feel I am good and not experiencing any severe problems with my health. I have no symptoms. Even if there is a minor problem, I can solve this by myself. Therefore, I have no concerns. At a later stage, if I must go to the doctor and check what the problem is, I will delay this moment until I really have to have the Pap test.” (*Participant 20; 48 years old, married*).*

#### Pro and cons of preventive behavior

The pros and cons of preventive behaviors like screening behavior were generally not well understood, and this resulted in a possible wrong reassurance of being perfectly well or cancer free:

*“My doubt was reduced after performing a Pap smear … I do not have any diseases … I have just completed my cervical checkup. I want to continue my remaining life!”* (Participant 25; 38 years old, married).

Overall, it was found that women had difficulties understanding the concept of preventive behaviors, as both pros and cons of preventive behaviors were reported as significant components of their intention to undergo cervical cancer screening.

### Self-care

Since most of the participants believed that self-care had a key role in preventive behaviors related to cervical cancer, this theme includes three categories, namely healthy lifestyle, stress management, and medical checkups.

#### Healthy lifestyle

There were mixed opinions related to the women’s abilities regarding cervical cancer-preventive behaviors, including healthy lifestyle and screening activities. A majority of women were concerned about their health and took a variety of general preventive measures. They believed that cervical cancer could be prevented by having a healthy lifestyle, including eating a balanced diet, performing regular physical activity, and avoiding environmental harms. Some women went on a diet to decrease their fat. One of them said:

*“In my opinion, the effective way against cervical cancer is having a healthy lifestyle. I have healthy diets such as daily fresh vegetables, fresh fruits, and low fat in my regimen. Moreover, I exercise at least for two hours, five times weekly.”* (Participant 21; 36 years old, married).

Another participant mentioned:

*“I indeed try to improve my eating behavior and take care of having low-fat diets ...”* (Participant 16; 32 years old, married).

The majority of participants mentioned they wanted to live without any problems; therefore, they took care of their health, especially in regard to their reproductive organs. One woman stated:

*“I understand that there are many reasons to live healthy. I try to care about my body, especially reproductive organs, in such a way that I can also enjoy my life.”* (Participant 21; 36 years old, married).

Another woman mentioned:

*“If I think something is bad in my health and genital organs, I will visit a physician … I don’t hesitate going to a physician at all, even to a male physician.”* (Participant 22; 36 years old, single).

Also, a woman said:

“*I believe that, for a healthy and well future, I have to perform everything that has an effective influence on my health, including eating healthy foods, performing daily physical activity, and having Pap tests because I love my life … A healthy life is important to me.”* (Participant 16; 32 years old, married).

#### Stress management

Some participants believed that stress could cause cervical cancer. Therefore, they applied different procedures, such as relaxation, listening to music, exercise, and positive thinking, in order to reduce their stress and fight against cervical cancer. As stated by one woman:

“*Yoga and sports make me relax. I think this helps me be powerful enough to fight cervical cancer.”* (Participant 25; 38 years old, married).

Another participant mentioned:

*“I regularly go to meditation twice a week … I also listen to music in the early morning and late at night. It makes me relaxed and calm … I think it will prevent me from developing [cervical] cancer.”* (Participant 18; 31 years old, single).

Positive thinking was mentioned to be suitable for cervical cancer prevention, as stated by one woman:

*“Having a balance between physical, emotional, and spiritual health is very important. This consists of exercising, eating, having spiritual beliefs, having and setting goals of daily life, etc.”* (Participant 18; 31 years old, single).

#### Medical checkups

Most of the screened participants in the present study achieved cervical screening services as part of a general female checkup planned by the Iran’s Ministry of Health and Medical Education. One woman said:

*“Although everyone has a vital role in his or her own health, the cervical screening service is part of a general female checkup planned by the Iran’s Ministry of Health and Medical Education (MHME). If MHME is no longer responsible for women’s health, other systems cannot help us.”* (Participant 20; 48 years old, married).

Some women placed more value on their health after the Pap test and took better care of their health for achieving a better life. As expressed by one woman:

*“I went to the doctor for a Pap test. I was so glad, and I forgot all my doubts and fears when the doctor informed me about my genital health.”* (Participant 18; 31 years old, single).

Overall, it was found that a healthy lifestyle, stress management, and medical checkups were important components of women’s self-care activities toward cervical cancer prevention.

### Religion and culture

Many women believed that religion and culture are important influences in preventive behaviors in regard to cervical cancer. This theme includes three categories, namely Islamic laws, cultural norms, and faith in God. As described below, there were mixed opinions related to the role of religion and culture.

#### Islamic laws

A common viewpoint was that Islam deeply persuades women to pay attention to their health, even though this means revealing their private parts to a physician. As one woman said:

“*Genital organs are the women’s identity. If I feel something is wrong with my genital organ, I just go to the female physicians. I don’t want a male physician inspecting my female organs at all, even if I die due to my religious beliefs and Islamic laws … According to Islamic law, showing genital organs to men (except your husband) is a great sin.”* (Participant 20; 48 years old, married).

Some women stated that even generally forbidden behaviors, such as drinking alcohol, were permitted by Islam. Rather than religion, cultural norms like chastity on the part of women described their reluctance to perform screening.

*“I believe that Islam permits drinking alcohol for treatment. You are even permitted to take some drinks that contain alcohol … Therefore, if such things are permitted by God, then He is definitely not going to penalize you.”* (Participant 17; 33 years old, married).

In contrast, another woman said:

*“Drinking alcohol is forbidden in the Quran and you should not drink it in any situations … Consumption is a major sin.”* (Participant 20; 48 years old, married).

#### Cultural norms

Culturally, the participants of the present study stated that the needs of their family members, especially their husbands, had priority over their own health, which in turn has an impact on cervical cancer screening.

*“In Islam, being a woman—and also being a mother—has a special value. In Iran, women also have commitments to their families. I believe that I need to do everything for everyone … I need to do things for my son … I need to take care of my son … take care of my husband … I need to do things for my husband ... My family members’ lives have* priority *over my own life.”* (Participant 24; 33 years old, married).

One woman said:

*“I’m willing to do regular checkups, especially when it concerns my female organs. However, I think male physicians do it more skillfully than female physicians. What is also important is that the doctor knows the Turkish language [Native language of the Zanjan people] so that I can share my problems with him.”* (Participant 23; 31 years old, married).

#### Faith in God

Women also mentioned that their religious views and their faith in God played an important role in preventive behaviors with regard to cervical cancer as well as toward safeguarding themselves against this cancer. For example, praying was obvious in participants’ statements. For many women, trust in God and praying had a major role in preventing cervical cancer. As one woman said:

*“I think that praying and reading the Quran are sources of relief and quietness. Praying and believing in God give me a peace of mind to care for my physical and mental health. I believe that it makes me feel spiritual, and it causes me to be more optimistic ... I think healing can be obtained from God.” (*Participant 16; 32 years old, married).

### Perceived social support

Based on the participants’ views, social, informational, and financial support were perceived as important components of preventive behaviors. Although some women recognized distress and worry about hearing the word “cervical cancer” and the negative consequences of screening behavior, others believed that receiving support from relatives helped them to improve their self-esteem and to reduce their doubts, and it encouraged them to perform preventive behaviors in regard to cervical cancer.

#### Social support

We asked about the positive effects of family members, such as husbands, regarding whether women are permitted or supported to undergo screening. A few participants mentioned other women whose husbands banned them from seeking and performing preventive behaviors like screenings. However, once it concerned them, the women reported that their family members supported them in performing screening behavior. As one of the women mentioned:

*“I have a good and supportive family. When I wanted to perform a Pap test, my husband accompanied me.* He always reminds me to perform it annually*. He says: I don’t like that you could lose your life because of cervical cancer. I would like you to be safe and well.”* (Participant 21; 36 years old, married).

One woman stated that the approval of her husbands was essential to performing preventive behaviors because of the cultural and religious context of Zanjan city. Having no supporters could negatively impact women’s screening acceptance.

*“Due to our culture [Zanjan city] … having the approval of our husbands is essential … The women here [Zanjan city] obey the orders of their husband. People always think a woman has to have a supporter, particularly having support from her husband …”* (Participant 17; 33 years old, married).

A few women reported that they received support from their friends. They could share their experiences about Pap tests with them. These participants were encouraged by friends to perform cervical cancer-preventive behaviors. In addition, peers could provide valuable information about cervical cancer prevention that might not have been received from general practitioners. A woman reported:

*“I’m ready to do a Pap test since many of my peers performed it regularly, and they encouraged me to perform it also. Persuasion by peers is very valuable ... Even more important than doctors …”* (Participant 17; 33 years old, married).

#### Informational support

Women also received information about cervical cancer and screening from mass media. As a 39-year-old screened woman said:

*“I was informed by a TV program that even some young females had cervical cancer or another type of cancer. After hearing this information, I was very scared, thus I decided to do a checkup. Anyway, as a woman, I’ve observed this cancer with a friend of mine. She had to take out her uterus and ovaries due to cervical cancer ... I found it essential to perform a checkup immediately to understand whether I was healthy or not …”* (Participant 21; 36 years old, married).

Media can also raise awareness and offer information on cervical cancer prevention plans. One participant reported that:

*“I feel more courage to do a Pap test after getting information from TV about a woman with developing cervical cancer. I know that the Pap test is essential for prevention and early detection of cervical cancer.*” (Participant 20; 48 years old, married).

Another woman mentioned:

*“I read in a paper on the Internet that healthy food and regular physical activity are effective for cervical cancer prevention. Consequently, I now often eat fresh fruits and vegetables, and I am on a low-fat regimen.”* (Participant 17; 33 years old, married).

#### Financial support

Having health insurance and a good economic condition are some of the helpful components that can persuade women to follow healthy behaviors. One woman said:

*“They [health care workers] can suggest Pap tests for free in the public hospitals and health care centers, right?”* (Participant 18; 31 years old, single).

A screened woman said:

*“I saw that some women didn’t get a Pap test because of their poor economic situation and lack of insurance … But I screened myself on time. It is too terrible to lose my health, my life, and my feminine organs [uterus and ovaries] because of money.” *(Participant 21; 36 years old, married).

### Perceptions of cervical cancer risk

This theme highlighted participants’ risk perception of cervical cancer and preventive behavior containing three categories, namely false risk perception, insufficient education, and misinformation.

#### Inaccurate risk perception

Risk perception was formed by a person’s private experiences about health, illnesses, and screening procedure as well as through his or her contact with family members and relatives in which any kind of cancer has been detected.

*“… In my relatives, I did not hear or see cervical cancer and prevention. I did not get any information about Pap tests … I only know this is just for cervical cancer detection! … There is no history of ovarian cancer or cervical cancer in my relatives, so why should I do this test unless I have any symptoms … I do not want to perform the preventive behavior like a Pap smear, due to the lack of any symptoms or problems.”* (Participant 20; 48 years old, married).

Most women believed that behaving immorally increased the risk for cervical cancer. However, there were some opposing opinions concerning multiple marriages. Islam allows men to marry four spouses at a time, and, based on Islamic law, each wife is legally married to the man. Although some women stated that cervical cancer is related to multiple sexual relationships, particularly with different persons, they did not think that being in a multiple marriage could enhance the risk of developing cervical cancer.

*“Islam lets men have multiple marriages [four wives] … It should help marital life if you know Islamic laws … Nevertheless, Islam pays much attention to the status of women in the family … about having a good relation with only one man for being safe and healthy.”* (Participant 25; 38 years old, married).

Only three women mentioned that multiple marriages enhanced the risk for cervical cancer. As one of them said:

*“Although Islam allows men to have four wives, if one of those four wives has a disease … the man is transmitting that disease … transmitting that disease to other partners; yes, unfortunately all four wives could catch it.”* (Participant 23; 31 years old, married).

The women generally described that most Muslim people were careful about being in multiple sexual relationships. Yet it is important to consider that the consequences of sexual intercourse were often understood in different ways. For instance, some women believed that if a woman was diagnosed with cervical cancer, it meant that she was a whore or she had sex with a married man. A common viewpoint was that immorality and sexual deviation in men were ignored, while female participants were held to greater ethical principles.

*“I believe that a woman has more of the fear of God in her … you know, really not to be defamed … not to be faulted … because if a woman is caught, the woman … is the one to be blamed, but the man goes free …”* (Participant 25; 38 years old, married).

Specific risky behaviors often mentioned by participants included having disloyal sexual partners, having too much sex, beginning sexual relations too young, having sexual contact during menstruation, and taking contraceptive pills. Many of the participants mentioned that once they started to have sex, they would be likely to get some kind of infection. Although the meaning of infection was dominant in their understanding of cervical cancer risk, they had no good knowledge about *human papillomavirus* as a pioneer to cervical cancer.

*“It is mostly a health problem, and this is related to sexual relationships. If your health status is bad, you are more likely to take a genital infection. If the infection becomes worse, it will develop into cervical cancer. Cervical attritions can develop into a certain level, maybe modest or severe; at the very beginning, it might be a precancerous sore.”* (Participant 23; 31 years old, married).

Overall, the women provided a fairly low rating to their own risk of developing cervical cancer. Some women perceived a low risk due to not having any symptoms or having no family history of cervical cancer. On the contrary, participants with a family history of cervical cancer or any other cancer perceived themselves at an increased risk for cervical cancer. However, these women also believed that the increased risk could be offset, as they had no physical symptoms themselves. A non-screened woman with a family history of cervical cancer justified her perceived risk of cervical cancer by saying:

*“Risk is linked to family history. My physician also highlighted that ... That shows I am at a disadvantage. But I am 31 years old, and at this moment, I have no signs of cervical cancer.”* (Participant 27; 31 years old, married).

Almost all women, including those who had never been screened, received information about cervical cancer and preventive behaviors. They were informed that cervical cancer can be prevented and treated. Fifteen out of the total women who were interviewed had received information about cervical cancer and prevention from the health education presented at the health care centers and clinics.

*“Indeed, I have received information about cervical cancer, which is one of the sexually transmitted illnesses. Therefore, it is reasonable to prevent this disease [cervical cancer] … If you undergo screening early enough … It becomes easier to prevent it. If they detect that you are healthy, they recommend which preventive methods you have to perform.”* (Participant 18; 31 years old, single).

#### Insufficient education

Although most women received information about cervical cancer and prevention from the health education at health care centers and clinics, there were complaints about insufficient education regarding cervical cancer and preventive methods. Almost all participants described that health education was not easily presented, had gaps, and was not organized well. For instance, the majority of participants described that the time provided to health education discussions in health care centers was very brief and, as such, that many topics about cervical cancer were not sufficiently described to them. They mentioned that the health staff only recommended cervical screening facilities to them and persuaded them to perform Pap smears without providing regular and in-depth information about the cervical cancer and preventive behaviors or the related facilities. The women believed that this was due to the heavy responsibility combined with the employees’ insufficiency.

*“The health discussions and programs that are shown here at the health care centers are in intervals. So if someone comes very soon, she can achieve some health discussions, but others who come late … they achieve no knowledge about prevention of cervical cancer … Yeah … not knowing about risk factors of this disease and anything else …”* (Participant 20; 48 years old, married).

Another woman said:

*“… The health care employees just said to us that we should perform screening, but they did not mention much about the prevention of this cancer and existent approaches. They didn’t give us more details and information. I was pregnant, and I did not know whether it was acceptable to screen during my pregnancy.” (*Participant 25; 38 years old, married).

Two experts also agreed with the women that education about cervical cancer and preventive behaviors was not enough. They described that the short period of education did not provide a chance for all women to receive correct information about the cancer. There are many subjects to deal with during education. The health educational programs are performed only in the morning due to the health care centers’ working hours, and the people who cannot participate during this time miss the programs. Furthermore, they mentioned that educational programs about cervical cancer are not continuously performed and that there is not enough time to address all topics because of the high responsibility of health care workers.

*“Unfortunately, I know that health educational programs about cervical cancer are not commonly performed, depending on the amount of health care staff members in health centers [in Zanjan] … Not all health care staffs [in our city] are well informed about cervical cancer information … Therefore, not just anyone can choose to participate in an educational program and discuss this disease and preventive behaviors … So when we do not have enough health care staffs [in Zanjan] in the reproductive health, then the educational programs about this cancer will not be properly implemented in our city.”* (Health care worker; 34 years old, married).

#### Misinformation

A few women who had previously undergone a Pap test did not believe they had to undergo it regularly. Those who had entered the screening program mentioned they had not been provided a suitable reason as to why they had to perform screening once more. They just did it regularly as part of the self-care program in health care centers [in Zanjan].

*“For me, I undergo the screening [for cervical cancer], but there is not anything [suitable data] … the doctor said to me that I may have cervical cancer due to the history of cancer in my family, but I feel that maybe I don’t need to perform it again and once more.”* (Participant 20; 48 years old, married).

As another woman said:

*“I don’t have any information about how cervical cancer is prevented … maybe they give drugs … or … maybe they give vaccination. I actually don’t know anything about the preventive process of this cancer … health care workers told me when I performed a Pap test, if it is detected that I have this cancer, I will be required to take some medicines … but I did not receive any correct information about medicines … I don’t know how the cancer is treated … how it is prevented. This is awful …”* (Participant 18; 31 years old, single).

## Discussion

The aim of the present study was to explore women’s experiences regarding cervical cancer-preventive behaviors. As a result, six main concepts emerged, including attitudes toward cervical cancer and preventive behaviors, preventive behaviors’ concept, self-care, religion and culture, perceived social support, and awareness about cervical cancer and preventive behaviors.

Preventive behaviors can be defined as behaviors expected for the prevention or early detection of diseases like cervical cancer at an asymptomatic stage [40]. These behaviors are not equally revealed among social groups. However, studies have demonstrated that women who are in social class 5 (those most at risk of developing cervical cancer) are least likely to perform cervical cancer screening. Studies have also revealed that women who are not interested in screening may be older, have a lower salary, or come from a lower socioeconomic class than those who undergo a Pap test [40, 41].

Evidence shows that having interest and positive attitudes in remaining healthy may encourage women to perform preventive behaviors like Pap tests against cervical cancer [36, 42]. Our findings emphasize that social, cultural, and religious factors influence women’s cervical cancer-preventive behaviors and that there are various misconceptions and superstitious beliefs among women. Many women mentioned that they were scared of the word “cervical cancer,” and some said that even talking about it might cause cervical cancer. These findings are consistent with another study revealing that there are fears, misconceptions, and fatalism among women [43]. The present study also revealed that some participants believed in legends regarding the causes for cervical cancer and, as such, were not well informed. It seems that providing correct and appropriate data and good educational programs might resolve these misconceptions and fears about cervical cancer.

Similar to other studies, some women in this study found the Pap test to be a shameful process [42–45]. Women who had never been screened also reported fear, shame, and embarrassment as barriers to preventive behaviors. It was obvious in the current study that shame was a worry among Iranian women, once again highlighting the influence of cultural norms. It is vital to decrease these fears and shames to increase cervical cancer screening. Attitudes and beliefs may be valuable tools in designing interventional programs to encourage women to change their unhealthy lifestyles and adopt more positive attitudes toward preventive behaviors regarding cervical cancer.

Similar to other studies, some participants expressed worries related to their martial relationship [42]. These women thought that developing cervical cancer is like losing their marital relationship and femininity. They mentioned that reproductive organs are a symbol of femininity and sexuality. Such beliefs about cervical cancer seem prevalent among several women worldwide. Moreover, different studies have described that women are very worried about their reproductive and genital organs, as they believe genital organs shape their feminine identity [42, 46–48]. Health care workers might take this into consideration as a reinforcing factor to encourage women to see their physicians when they feel something is incorrect with their health.

The majority of women mentioned that self-care is a key factor to perform preventive behaviors related to cervical cancer. They believed that having checkups based on the guidelines of the Iran’s Ministry of Health and Medical Education and having a healthy lifestyle such as eating fresh vegetables and fruits, exercising regularly, and controlling weight will help them prevent cervical cancer. We believe these are good indications that women are becoming more aware of the factors that promote cervical cancer and that they are ready to modify their lifestyles if health care workers identify such key abilities for behavior modifications among women.

We discovered mixed results with regard to the relationship between stress and cervical cancer. Similar to some studies [49–51], our findings indicated that many women believed that external stress was related to cervical cancer and that using relaxation, music, exercise, and positive thinking might reduce their stress. Stress management might not only enhance women’s health but also increase preventive behaviors toward cervical cancer [52].

Overall, our study highlighted the possible positive role of religion and culture in influencing cervical cancer-preventive behaviors and promoting health among women. Some women mentioned that their religious and personal priorities involved visiting female physicians, which was similar to other studies performed among Muslim women [44, 45]. Furthermore, our findings support the idea that religious beliefs may offer effective coping procedures. In the present study, some women emphasized that their faith and religious actions offer valuable approaches for managing stress (e.g., by reading the Quran and by praying) and that religious beliefs can be an important source of support during difficult periods. Surrendering control to God was observed as a vibrant and careful method that improves mental health by not focusing on features of cancer that are beyond the individual’s control.

Some participants also mentioned they had more trust in the skills of male physicians. This is another possible cultural effect. Some women also showed preference toward religious physicians. However, overall, religion was not the main factor when seeking a physician. Many women asked for Turkish-speaking doctors (Zanjanian people speak Turkish), because they believed these doctors are more skilled. Yet no clear priority was declared for any specific ethnicity in relation to doctors. The ability to talk and link with eligible and compassionate doctors in their native language was very important to the women. Other studies have not shown the effect of the doctor’s ethnicity and religion in relation to cervical cancer screening.

In the present study, we found that cultural opinions about cervical cancer could cause worry, and this worry could result in behavioral reactions such as having no interest in performing screening behaviors and even avoiding seeking correct information about the nature of cervical cancer and preventive behaviors. Furthermore, having various levels of financial, emotional, and informational supports from different sources encouraged women to perform preventive behaviors regarding cervical cancer. Regarding perceived social norms, the present findings revealed that most of the supports were offered by family, especially by the spouse. In fact, family is the first and most key source of receiving support for performing healthy behaviors [53]. Studies have also demonstrated that receiving support from family members, doctors, and friends might lead to performing preventive behaviors and screening uptake [54–56].

An overall lack of awareness among participants about cervical cancer and screening behavior is an important finding of the present study. Participants had incorrect risk perceptions and found that the educational program was insufficient and sometimes provided false information. This finding is consistent with other studies in which women did not follow cervical cancer-preventive behaviors due to a lack of correct information about that nature of that disease [56, 57].

Similar to other studies [8, 10], our findings revealed that women believed that the risk of having cervical cancer was influenced by a family history of cervical cancer and that if there was no cervical cancer among their family and there were no symptoms, therefore, they did not need to perform a Pap test. Although the women mentioned that cervical cancer had severe consequences, many of them did not perceive themselves to be at risk for it. In a way, our findings also show a discrepancy between the perception of cervical cancer’s severity and its risk. This is because cervical cancer is sometimes considered shameful and fatal. Some women believed that respect for morality and chastity would reduce the risk of cervical cancer and that if they behaved ethically and with chastity, they would not get cervical cancer. Not unexpectedly, some women were unwilling to openly claim they believe they themselves have a high risk of getting cervical cancer.

### Limitations

A limitation of this study is that we only interviewed women living in Zanjan; therefore, their views may not sufficiently represent the views of women living in other areas and within different cultures. In future studies, it might be interesting to conduct more qualitative interviews, combined with quantitative data collection, to investigate the experiences and desires of women from other regions and cultures.

## Conclusion

The present findings demonstrated that attitudes toward cervical cancer and preventive behaviors, preventive behaviors’ concept, self-care, religion and culture, social support, and correct awareness about cervical cancer and preventive behavior are key components that encourage women to perform (or not) cervical cancer-preventive behaviors.

## Abbreviations

DES: Diethylstilbestrol
FGD: Focus group discussions
HPV: Human papilloma virus infection
